# Refinement of atomic models in high resolution EM reconstructions using Flex-EM and local assessment

**DOI:** 10.1016/j.ymeth.2016.03.007

**Published:** 2016-05-01

**Authors:** Agnel Praveen Joseph, Sony Malhotra, Tom Burnley, Chris Wood, Daniel K. Clare, Martyn Winn, Maya Topf

**Affiliations:** a Institute of Structural and Molecular Biology, Department of Biological Sciences, Birkbeck College, University of London, Malet Street, London WC1E 7HX, United Kingdom; b Scientific Computing Department, Science and Technology Facilities Council, Research Complex at Harwell, Didcot OX11 0FA, United Kingdom; c Electron Bio-Imaging Centre (eBIC), Diamond Light Source, Harwell Science & Innovation Campus, OX11 0DE, United Kingdom

**Keywords:** Flexible fitting, Density fitting, Protein structure modelling, 3D-EM, Structure refinement, Model validation

## Abstract

As the resolutions of Three Dimensional Electron Microscopic reconstructions of biological macromolecules are being improved, there is a need for better fitting and refinement methods at high resolutions and robust approaches for model assessment. Flex-EM/MODELLER has been used for flexible fitting of atomic models in intermediate-to-low resolution density maps of different biological systems. Here, we demonstrate the suitability of the method to successfully refine structures at higher resolutions (2.5–4.5 Å) using both simulated and experimental data, including a newly processed map of Apo-GroEL. A hierarchical refinement protocol was adopted where the rigid body definitions are relaxed and atom displacement steps are reduced progressively at successive stages of refinement. For the assessment of local fit, we used the SMOC (segment-based Manders’ overlap coefficient) score, while the model quality was checked using the Qmean score. Comparison of SMOC profiles at different stages of refinement helped in detecting regions that are poorly fitted. We also show how initial model errors can have significant impact on the goodness-of-fit. Finally, we discuss the implementation of Flex-EM in the CCP-EM software suite.

## 1 Introduction

Cellular processes are governed by bio-molecular interactions, often forming large macromolecular assemblies. These assemblies are structurally stable but undergo conformational changes to support their functions. Structural characterization of such assemblies is essential for gaining insights into the vital cellular activities that they are involved in. Over the past decade, three-dimensional Electron Microscopy (3D EM) techniques have become widely accepted in solving structures of macromolecules involving different conformational states, in a close-to-native state [1]. However, in most cases, the low signal-to-noise ratio of these techniques still limits the determination of structures at atomic resolutions. Therefore, docking (or fitting) of atomic models (e.g., from X-ray crystallography, NMR or structure prediction methods) into 3D EM maps has become common practice with a rapidly increasing number of atomic models in the Protein Data Bank (PDB) associated with maps in the Electron Microscopy Data Bank (EMDB) (currently over 600) [2], [3], [4], [5].

Identifying the optimal fit of components is a challenging task, and it depends strongly on multiple factors including the map resolution, the accuracy of the atomic model of each component, and the scoring function used to measure the goodness-of-fit. Prior to density fitting it is necessary to locate the approximate positions of component structures. Localization of density of a specific component is often done by segmenting the density [6], which is followed by *rigid fitting* in six translation/rotation degrees of freedom to get the best configuration of the atomic model in the map [7], [8]. Another approach is multi-component fitting (*assembly fitting*), where multiple components are fitted simultaneously, making it a multibody optimization problem [4], [5], [9].

Once an atomic model has been fitted rigidly to a region of the EM map, it is often evident that its conformation is different from the one represented by the map. Such differences can arise from conformational changes upon its integration into a complex, different functional states of a complex, or a poor atomic model. To further improve the agreement between the model and the map *flexible fitting* methods can be applied. These methods vary in the representation of the atomic models, their flexibility, and the measure of goodness-of-fit with the EM map. To this end, several real-space refinement approaches have been developed [10], [11], [12], [13], [14]. These methods guide the conformational changes by optimizing the fit of the probe structure in the density while maintaining its geometric and mechanistic properties. For example, one of the earlier methods, RSRef [10] uses steepest descent or conjugate gradient shifts to improve the least-squares fit of the model and the map density. Flex-EM [11] optimizes a scoring function which includes cross-correlation with density as well as stereo-chemical and non-bonded interaction terms. YUP.SCX [15] approximates intra-molecular forces by a Gaussian Network Model (GNM) and the optimization protocol uses simulated annealing MD. In MDFF [12], external forces proportional to the gradient of the density map are combined with an MD protocol to optimize the conformations of the component structures. Flexible fitting approaches using normal mode analysis [16], [17] have also been developed, with the advantage of capturing large-scale conformational changes. In iMODFIT [18] for example, the lowest-frequency modes are randomly selected to generate trial conformations. Only those conformations that increase the cross-correlation (CC) with the density are accepted. Finally, DireX method utilizes a deformable elastic network (DEN [19]), defining springs between selected atom pairs from the reference model; as the refinement proceeds the network is adjusted to allow the equilibrium lengths to vary from the reference model if justified by the density. The conformational sampling algorithm is based on CONCOORD method [20] that uses distance restraints from the input structure and generates an ensemble of conformations that satisfy these restraints.

Although most EM structures are still at the low-intermediate range, in recent years, there has been a substantial rise in the number of high-resolution structures of assemblies solved by EM mainly due to the development of direct electron detectors [21] and improved image processing methods [22]. As a consequence of this so-called “resolution revolution” in this field, many crystallographic methods are being adapted for use with EM data [23], [24], [25], [26], [27]. Coot [23] is an interactive 3D modelling program designed for the building and validation of macromolecular structures largely based on crystallographic data. It includes the *Jiggle Fit* feature and the *model-morphing* tool which are designed for iterative local fitting to improve the agreement of model segments with the density. REFMAC [25] utilizes maximum likelihood to optimize model geometry and fit to the experimental data. Structure factors can be calculated for a local region of the EM density map of the assembly against which the component atomic model is refined. These features in Coot and REFMAC have been used for modelling atomic structures of ribosomes in EM density maps better than 4 Å [28]. Recently, Rosetta has been applied to fit models in high-resolution density maps [29]. Selection and optimization of fit of local backbone fragments are carried out using a Monte Carlo approach with constraints applied to maintain the backbone and side-chain geometry. The fragments are then stitched together and energy minimized to regularize overall geometry.

Both rigid and flexible fitting result in an atomic model, for which the quality assessment is not trivial. Approaches that try to address this issue include the use of confidence intervals, quantifying the best-fitting model relative to a distribution of different fits, local scoring (using a mask) and the use of multiple scores [30], [31], [32], [33], [34], [35], [36]. It is also common practice to validate the refined model against an independently reconstructed density map (cross validation) [37], [38], [39]. We have recently used local assessment of flexible fits at different levels of structure representation (domains, secondary structure elements and loops) [40], [41], [42], [43] as implemented in TEMPy [30]. This software is useful for both density map and atomic structure processing and fit assessment (model-to-map and map-to-map), especially in the intermediate-to-low resolution range.

The Flex-EM method we have previously developed as part of MODELLER [11] was designed originally to refine models at the intermediate resolution range (5–15 Å). It was applied successfully to several protein complexes, including the elongation factor EF4 bound to the *Escherichia coli* ribosome at 11 Å resolution [44], the apoptosome-procaspase-9 CARD complex at 9.5 Å [45], multiple intermediates of ATP-bound GroEL at 7–9 Å [46], microtubule bound kinesin motor domain at 7 Å [42], and the 11 Å liposome-embedded 13-fold pleurotolysin [43]. Here we present a few test cases where Flex-EM is used to refine models in high-resolution EM density maps (2.5–4.5 Å). The use of rigid body (RB) restraints and local assessment of the model during the course of fitting are highlighted. We also discuss the implementation of Flex-EM as part of the Collaborative Computational Project for Electron cryo-Microscopy (CCP-EM) [47].

## 2 Theory/calculation

We examined the use of Flex-EM for refining structures of three proteins in density maps of resolution better than 5 Å, three experimental (Sections 3.1.2, 3.1.3 and 3.1.4) and one simulated (to 3 different resolutions, section 3.1.1). In two cases the initial models were obtained by homology modelling using MODELLER [48] and in the other two cases (Sections 3.1.3 and 3.1.4), we used the crystal structure of the protein in a different conformation. In all cases, the initial models were first fitted rigidly using the *Fit-in-Map* tool in Chimera [49], where the CCC (Cross-Correlation Coefficient) score of the fit is maximized, and then subjected to Flex-EM refinement.

For the three cases where experimental maps were used, we performed flexible fitting of a component model into a segmented density corresponding to the location of the component in the map of the molecular assembly. To minimize additional density corresponding to other components of the assembly, segmentation was carried out first in a stepwise manner, using Segger implementation in Chimera [6], [49]. Initially, the map of the full assembly was contoured using a relaxed threshold. The contoured maps were then segmented into regions smaller than the size of the component. Those segments encompassing the component were manually grouped together in Chimera to extract the density. This was followed by a final step of segmentation where the extracted density was further divided into segments that are smaller than those in the previous step. Again, segments were selectively grouped to avoid those that are not in the immediate vicinity of the initial model.

Chemical and structural restraints based on prior knowledge can help to preserve the correct geometry in cases where local structures would otherwise be distorted during refinement and also reduce over-fitting. The RIBFIND method identifies RBs by treating α-helices and β-sheets as basic units and clustering them based on their spatial proximities [50]. Following that, the group of secondary structural elements (SSEs) in each cluster and the loops connecting them are treated as one RB. SSEs that do not fall into any cluster are treated as independent RBs.

Flex-EM conformational refinement was performed using a heuristic optimization that relies on simulated annealing molecular dynamics (MD) applied to a series of subdivisions of the structure into progressively smaller RBs [11]. During the refinement, the coordinates of the RBs into which the structure is dissected are displaced in the direction that maximizes their cross-correlation with the EM density map and minimizes the violations of the stereochemical and non-bonded terms [11]. The stereochemical restraints include (a) a harmonic potential with the mean equal to the value in the current structure and a force constant typical for chemical bonds, angles, and improper dihedral angles obtained from a parameter library that uses the CHARMM22 force field [51]; and (b) the two-dimensional (ϕ,φ) dihedral-angle restraints based on the Ramachandran plot [52]. The non-bonded term is a sum of the harmonic lower bounds (left Gaussian) of all nonbonded atom pairs; the lower bound is the sum of the two atomic van der Waals radii [51], and the force constant is 400 kcal/mol/Å^2^. The system is described by individual atoms and/or RBs (groups of residues), upon which the forces act. The temperature of the system is gradually increased from 0K to 1000K and then decreased back to 0K. Multiple cycles of simulated annealing may be required for the convergence of global correlation, which is provided in the output from each run.

**Fig. 1:**
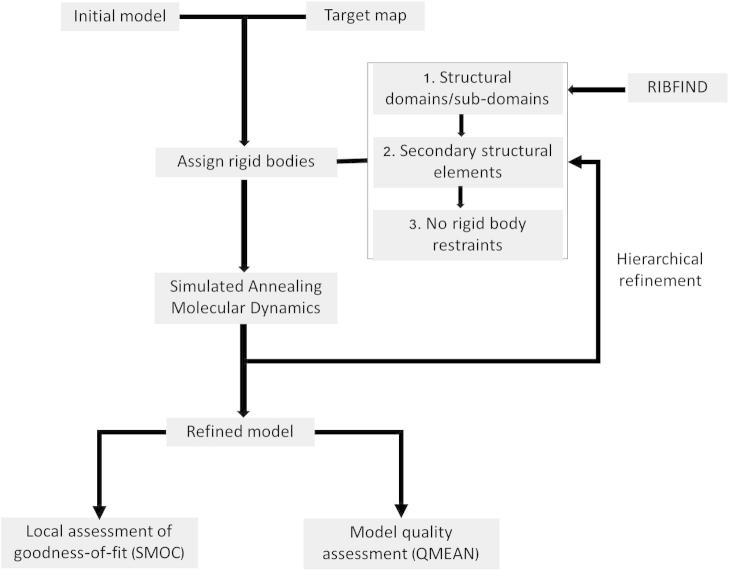
Workflow for flexible fitting with Flex-EM and assessment of the fitted model.

Flex-EM runs were carried out in a hierarchical manner by considering RBs at different levels: domains/sub-domains identified by RIBFIND and then in the next stage, individual SSEs (Fig. 1). For large body motions, the maximum atom displacement along one axis at each MD step is limited by 0.39 Å [11]. In cases where the model does not have multiple domains/sub-domains that can be identified as large RBs (first two examples below: Sections 3.1.1 and 3.1.2), we started by constraining SSEs as RBs. At this stage, we adjusted the protocol for the high-resolution density, with maximum shifts of all atoms limited to 0.15 Å at each MD step. Also, at the final refinement stage, relative motions between all atoms were allowed without considering any RBs. The maximum atom displacement steps were reduced to 0.1 Å in this final stage for finer fitting refinement.

In order to assess the model agreement with the density locally we used a score similar to the segment-based cross correlation score (SCCC) in TEMPy [30] (Fig. 1). Instead of the standard cross-correlation coefficient involving deviation from the mean, we calculated the fit of a segment of residues ‘s*r*’ using the Manders’ Overlap Coefficient [40], which is closely related to CCC:

$$\mathrm{SMOC}=\frac{\sum_{i\in \mathrm{vox}\_\mathrm{sr}}\rho_{i}^{\mathrm{EM}}\times \rho_{i}^{P}}{\sqrt{\sum_{i\in \mathrm{vox}\_\mathrm{sr}}{\left(\rho_{i}^{\mathrm{EM}}\right)}^{2}\times \sum_{i\in \mathrm{vox}\_\mathrm{sr}}{\left(\rho_{i}^{P}\right)}^{2}}}$$

where vox_sr indicates all voxels in the density grid that are occupied by atoms in the segment. $\rho_{i}^{\mathrm{EM}}$ represents the target map density at the grid point ‘*i*’ and $\rho_{i}^{P}$ represents the model derived density at ‘*i*’. A similar score is also implemented in Chimera [49] for assessing fits of selected regions in the model. Generally, it is less sensitive to the position of the mean in the density distribution, especially in cases where the target map contains density additional to that represented by the component being fitted. Also, it is not influenced greatly by the differences in intensities between the components of the map [40].

Segment-based Manders’ Overlap Coefficient (SMOC) was calculated for each SSE, and the connecting loop residues were scored over overlapping windows of nine residues. For assessing model quality, we used Qmean score [53] which uses a knowledge based potential described by a scoring function that evaluates distance dependent all-atom and residue interaction potentials, torsion angles, solvation and agreement with predicted secondary structure and solvent accessibility [53].

Currently Flex-EM is distributed as a downloadable set of python scripts that run with MODELLER [48]. We have developed a user-friendly interface as part of the Collaborative Computational Project for Electron cryo-Microscopy (CCP-EM) [47], which integrates Flex-EM with RIBFIND for identifying sub-structures that can be treated as RBs. The interface also facilitates user intervention to decide the input parameters, enables interactive visualization of selected RBs and fitted models, and outputs plots on the model fitting scores. In order to give a consistent look-and-feel the Flex-EM interface uses the same toolkit as other software in CCP-EM.

## 3 Results

### 3.1 Application of Flex-EM refinement protocol to high-resolution 3D-EM maps

Below we describe the results of refinement of four different examples, one against density simulated from a protein crystal structure in PDB [54] and the other three against experimental maps (two taken from EMDB [2] and one is a newly reconstructed map). We discuss the results of these tests and potential improvements to be incorporated for application in this range of resolutions.

#### 3.1.1 *E. coli* adenylate kinase (2.5 and 3.5 Å resolution)

**Fig. 2:**
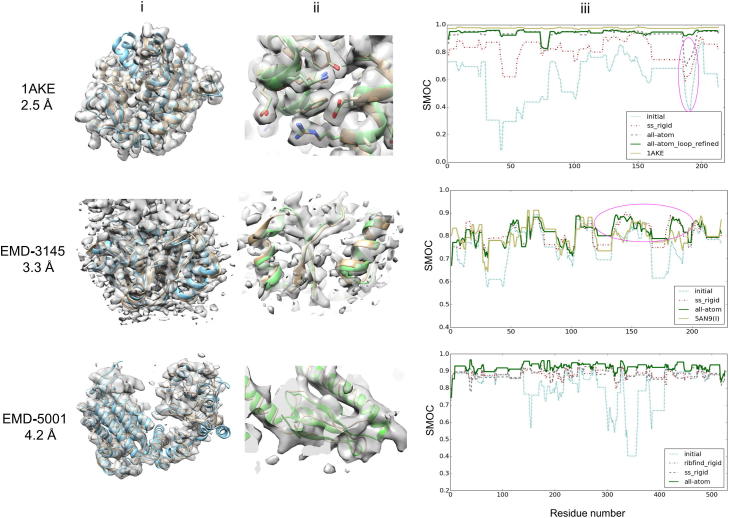
Flex-EM refinement at high-resolution. The top row corresponds to refinement of a homology model of *E. coli* adenylate kinase in a 2.5 Å resolution density map representing an inhibitor-bound form (PDB: 1AKE). The middle row shows the refinement of a homology model of Initiation factor 6 (eIF6) into a 3.3 Å resolution map of 60S ribosome from *D. discoideum* bound to eIF6 (EMD-3145). The bottom row depicts the flexible fitting of ADP-bound GroEL (PDB: 4KI8) to the density corresponding to the unliganded form solved at 4.2 Å resolution (EMD-5001). For these three examples, we show (i) a comparison (using Chimera) of the starting model (blue) with the X-ray or deposited model (light-brown) associated with the density map (transparent grey); (ii) a comparison of side-chains or backbone in a small region of the refined model (green) with those of the X-ray structure or the deposited model (light brown) associated with the map; and (iii) SMOC profile showing scores in each stage of Flex-EM run.

A 2.5 Å resolution density map was simulated from the crystal structure of *E. coli* adenylate kinase bound to an inhibitor (PDB: 1AKE). A different conformational state of the protein was modeled using a template structure of a mutant adenylate kinase from *Saccharomyces cerevisiae* (sharing 46% sequence identity), bound to an ATP-analogue representing the closed state (PDB: 1DVR). This homology model has Cα and all-atom RMSDs of 4.53 and 4.73 Å from the original *E. coli* conformation (1AKE), respectively (Table 1, Fig. 2(i)). Flex-EM real-space refinement was aimed at refining the model in the closed state based on the density map of the transition state.

For the initial Flex-EM run, the SSEs were constrained as RBs. The global CCC flattened after two iterations of simulated annealing. Analysis based on SMOC also showed improvement in most regions along the protein chain (Fig. 2(iii)). Next, the model was refined further, but this time all atoms were allowed to move throughout a single iteration of annealing.

The SMOC profile of the model from the first iteration of all-atom refinement stage reflected a global improvement in the model fit (Fig. 2(iii)). However, segments 185–193 and 74–80 had poor scores compared to the rest of the model and showed minimal improvement over different stages of refinement. Segment 185–193 corresponds to a loop at the C-terminal end of a helix and segment 74–80 involves a 3_10_ helix.

**Fig. 3:**
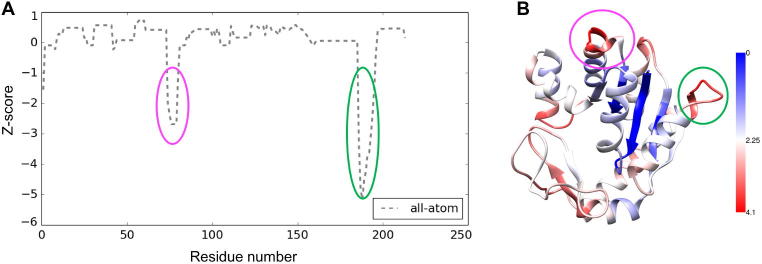
Refinement of a model of *E. coli* adenylate kinase in a 2.5 Å resolution simulated density map in inhibitor-bound form (PDB: 1AKE). (A) Z-scores calculated based of SMOC profile for the model obtained from all-atom Flex-EM refinement. (B) Starting homology model for fitting (using a homolog from *S. cerevisiae*) colored by local residue errors based on Qmean scores (as indicated in the side bar) using Chimera. Residue segments 74–80 and 185–193 with significant (and low) Z-scores, are highlighted in pink and green respectively.

We next calculated Z-scores based on the SMOC profile of the model generated after all-atom refinement (Fig. 3A): Z=(sr-μ)/σ where *sr* is the SMOC score associated with each residue and *μ* and *σ* refers to the mean and standard deviation of SMOC scores of all residues. As expected, two segments had significantly lower scores: loop 185–193 and segment 74–80 involving a 3_10_ helix. Analysis of the local residue errors in the starting homology model based on Qmean scores [53] (Fig. 3B) showed that the residues in both segments (highlighted in pink and green) had high residue errors, reflecting poor model quality.

**Supplementary Fig. 1:**
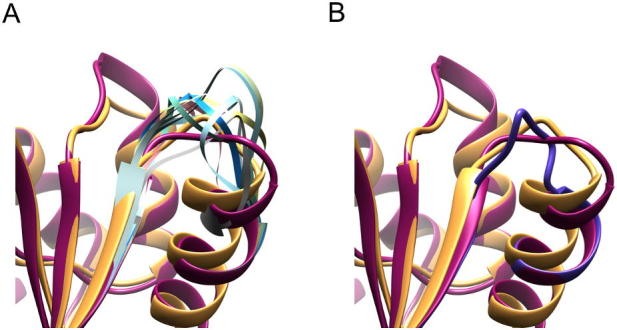
Refinement of loop 185–193 of E. *coli* adenylate kinase homology model in a 2.5 Å resolution map representing an inhibitor bound form (PDB: 1AKE). (A) Alternative conformations generated for segment 185–193 with MODELLER are shown as flat ribbons. The X-ray structure (1AKE) is colored in gold and the selected loop conformation for refinement in magenta. (B) Colors as in (A), the initial model from the SSE-based refinement is in purple.

To check whether the starting conformation of the loop 185–193 was the limiting factor for refinement of this region, we sampled 200 conformations for this segment with MODELLER loop refinement method [55] (Fig. S1). The SMOC scores for this segment were calculated and the top-scoring model was chosen. The all-atom refinement was carried out again with the loop-refined model and the SMOC scores showed significant improvement in this loop segment as well (Fig. 2(iii), marked in pink).

**Supplementary Fig. 2:**
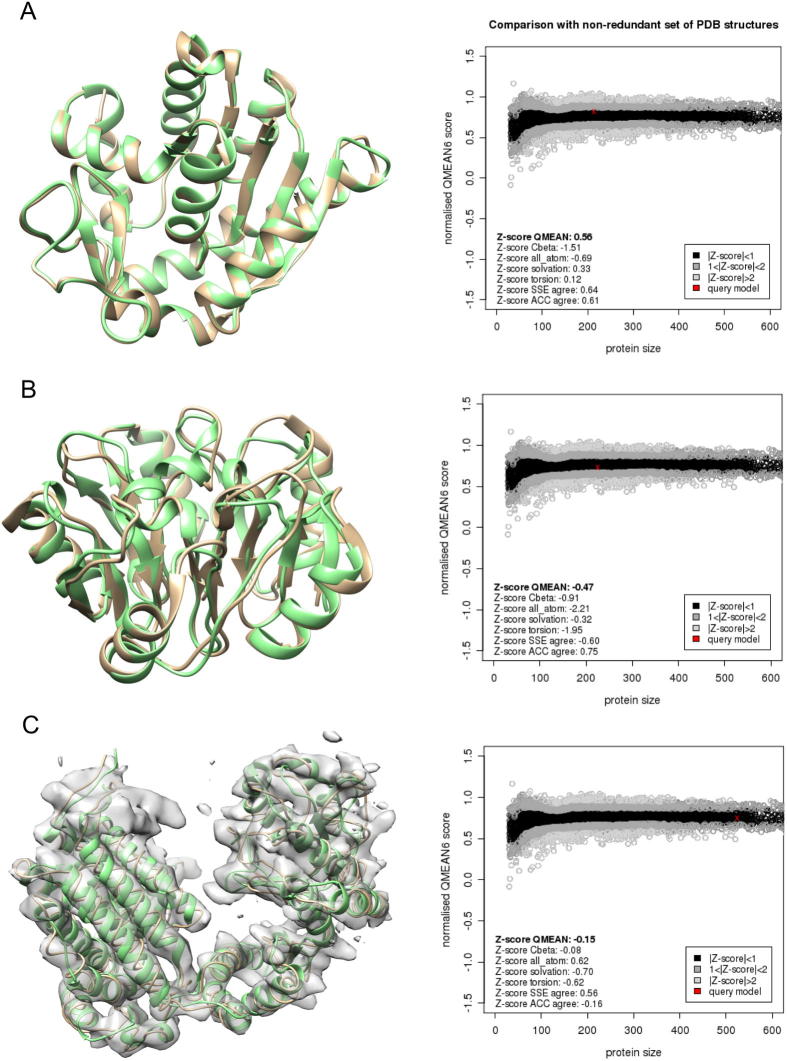
(A) Refinement of a homology model of *E. coli* adenylate kinase homology model into a 2.5 Å map representing inhibitor bound form (1AKE). Left: comparison of the refined model (green) with the X-ray structure (light-brown). Right: Qmean analysis (http://swissmodel.expasy.org/qmean/cgi/index.cgi) of the refined model showing the agreement with high quality structure in PDB. (B) refinement of a homology model of Initiation factor 6 (eIF6) into a 3.3 Å resolution map of 60S ribosome from *D. discoideum* bound to eIF6 (EMD-3145). Left: comparison of refined model (green) with the X-ray structure (light-brown). Right: Qmean analysis of the refined model showing the agreement with high quality structure in PDB. (C) Flexible fitting of ADP-bound GroEL (PDB: 4KI8) to the density corresponding to the unliganded form solved at 4.2 Å resolution (EMD-5001). Left: comparison of the refined model (green) with the X-ray structure (light-brown). Right: Qmean analysis of the refined model showing the agreement with high quality structure in PDB.

The final refined model had Cα and all-atom RMSDs of 0.41 and 0.96 Å, respectively, from the X-ray structure (1AKE) (Fig. S2A). The SMOC profile showed that the scores were similar to that of the X-ray structure in most regions along the protein chain. Visual inspection confirmed that most of the side-chains fit well in the density (Fig. 2(ii)) and assessment using the Qmean score suggested that it conforms with standard geometries and residue interaction potentials observed in high-resolution structures deposited in PDB (Fig. S2A).

To further validate our refinement method we performed a reverse experiment, by refining the crystal structure of *E. coli* adenylate kinase bound to an inhibitor (PDB: 1AKE) against a 2.5 Å resolution map representing the ATP-analogue bound form (which was generated from the homology model based on 1DVR). Without performing the loop optimization analysis that we described above, the final Cα and all-atom RMSDs with respect to the target model were 0.98 and 1.54 Å respectively. The Cα and all-atom RMSDs in the original experiment were very similar (0.89 and 1.44 Å, respectively), reflecting the robustness of the method.

Finally, we repeated the same steps of refinement of the original homology model into simulated maps at lower resolutions – 3.5 Å and 4.5 Å – from 1AKE. For refinement in the 3.5 Å map, the loop region corresponding to residues 185–193 was refined in a similar way to 2.5 Å and the highest scoring conformation was selected for a final all-atom Flex-EM refinement. However, in the 4.5 Å map based refinement, we chose a shorter loop segment (residues 186–192) based on the SMOC Z-score profile for the local conformational sampling with MODELLER. For the 3.5 Å map, the final fitted model has Cα and all-atom RMSDs of 0.64 and 1.19 Å respectively, while the model refined based on the 4.5 Å map has Cα and all-atom RMSDs of 1.09 and 1.76 Å, respectively (Table 1).

#### 3.1.2 *Dictyostelium discoideum* eIF6 bound to 60S ribosomal subunit (3.3 Å resolution)

A homology model of *D. discoideum* eIF6 based on the X-ray structure of the Initiation factor 6 (eIF6) from *Methanocaldococcus jannaschii* (sharing 36% sequence identity), was refined in a 3.3 Å map of eIF6 bound to 60S ribosomal subunit from *D. discoideum* (EMD-3145) [56]. For comparison we used the model fitted into the density map deposited in PDB (PDB: 5AN9; eIF6 model has the chain ID ‘I’). The map was pre-filtered before EMDB deposition, to remove the dust. We contoured it to include all the densities beyond the background peak and segmented eIF6 using Segger implementation in Chimera [6], [49] (Fig. 2(i)). About 23% of the map density is additional when compared to the volume expected to be occupied by the model. The Cα and all-atom RMSDs of the initial model from the fitted model are 1.9 Å and 2.9 Å, respectively (Table 1).

**Supplementary Fig. 3:**
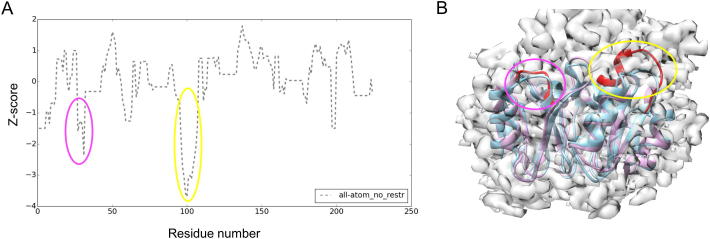
(A) Z-scores calculated based of SMOC profile for the model obtained from all-atom Flex-EM refinement without interface RB restraints. (B) Residue segments 95–106 and 26–32 with significantly low (<−2.0) Z-scores, are highlighted in yellow and pink respectively. The target density map (3.3 Å resolution map of 60S ribosome from *D. discoideum* bound to eIF6 (EMD-3145)) is shown in transparent grey.

At the initial stage of the Flex-EM run, the secondary structures of the model were constrained, followed by an all-atom refinement (Fig. 1). A single iteration of simulated annealing MD was sufficient to reach the best global CCC and the SMOC profile improved overall along the protein chain (Fig. 2(iii)). eIF6 has a β/α propeller fold that forms a strong interface with 60S ribosomal proteins L23 and L24 through a set of five pseudo-symmetric loops forming the interacting surface. These exposed interface loops were refined in the initial run while the SSEs were kept rigid. At the all-atom refinement stage, these loop regions were considered rigid, to prevent over-fitting via movement of atoms into the density corresponding to the interacting partners. In the absence of RB restraints at the interface, loop segments (especially residues 95–106 and residues 26–32) began to move into the partner density (Fig. S3) and this is reflected as areas of significant decline in SMOC scores (Fig. S3A).

The final refined model has Cα and all-atom RMSDs of 1.5 Å and 2.5 Å from the original fitted model deposited with the map (Figs. 2(ii) and S2B). The SMOC plot shows that the scores improve especially towards the C-terminus of the chain (Fig. 2(iii)). Visual inspection of density fit in this region (residues 120–180) suggests that the backbone fits better into the density than the fitted model deposited with the map (Fig. 2(ii)). Assessment of the final model quality with the Qmean score suggests that the model satisfies the standard torsion angles as well as residue and all-atom interaction potentials observed in proteins of similar lengths (Fig. S2B).

#### 3.1.3 GroEL (4.2 Å resolution)

The structure of the GroEL subunit is characterized by equatorial, intermediate and apical domains, and the binding of ATP/ADP to the intermediate and equatorial domains results in large conformational differences [46]. In this example we refined ADP-bound GroEL (PDB: 4KI8) in the experimental density corresponding to the unliganded apo-form solved at 4.2 Å resolution (EMD-5001). The map is associated with a fitted Cα-only model (PDB: 3CAU, chain A), which has a Cα RMSD of 3.59, 6.95 and 8.28 Å from the ADP-bound form, for the equatorial, intermediate, and apical domains, respectively. Prior to segmentation, a threshold level of 0.1 was used to contour the map. Although this level (<0.5σ) is quite low compared to the contour level of 0.6 recommended by the authors (density ranges from 0.006 at background peak to 2.42), it was chosen to avoid most of the discontinuous densities that begin to appear closer to the background peak. The map corresponding to a single subunit was segmented using Segger implementation in Chimera [6], [49] (Fig. 2(i)). The segmented density had about 44% additional density compared to that expected for the model.

**Supplementary Fig. 4:**
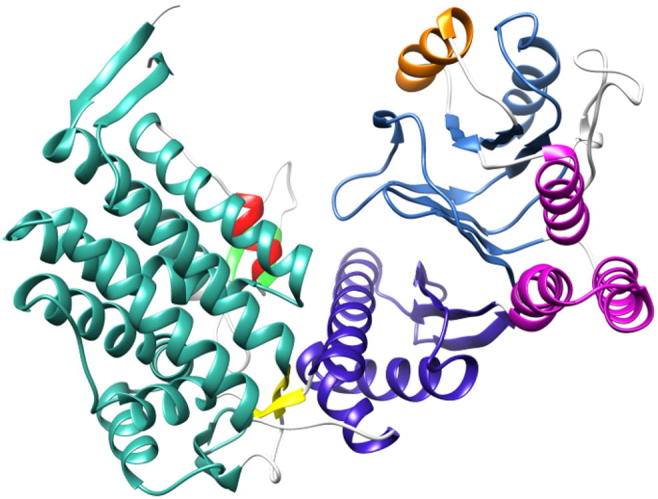
Rigid bodies (in different colors) identified by RIBFIND for the ADP bound GroEL structure (PDB: 4KI8) using a 32% cluster cutoff for merging secondary structures into rigid bodies [57]. White indicates regions that are not clustered.

Flex-EM fitting refinement was performed in multiple stages. In the first stage, large RBs identified by RIBFIND [57] (Fig. S4) were restrained to enable large body movements towards the density. After five simulated annealing iterations, the backbone fits into the density and the SMOC profile improved for the apical and intermediate domains compared to the initial state (Fig. 2(ii, iii)). At the next stage, SSEs were kept rigid and this was followed by an all-atom refinement (Fig. S2C). Qmean analysis of the model suggests that the interaction potentials and torsion angles confirm to standard ranges observed in high quality structures in PDB. The refined model has quite significant variations from the model deposited with the map, with Cα RMSDs of 4.53 Å, 5.36 Å and 5.19 Å for the equatorial, intermediate and apical domains.

#### 3.1.4 GroEL (3.3 Å resolution)

To compare model refinements carried out on raw and sharpened maps, we used an *E. coli* GroEL map in the apo-form, reconstructed at 3.3 Å resolution (Suppl methods). The raw density map was sharpened by applying a negative B-factor of −105 Å^2^, that was estimated using automated procedures [58]. As in the previous section, we used ADP-bound GroEL (PDB: 4KI8) as the starting model and followed a similar hierarchical refinement procedure as in Section 3.1.3. In this case we refined the model separately in the raw and processed maps.

**Supplementary Fig. 5:**
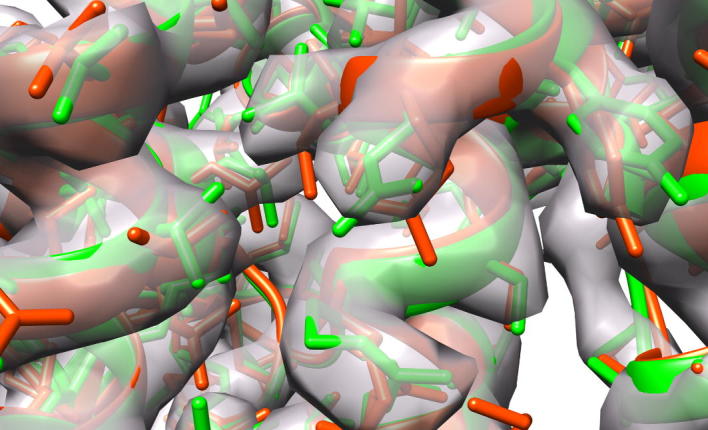
Flexible fitting of ADP-bound GroEL (PDB: 4KI8) to raw and sharpened density maps corresponding to the unliganded form of GroEL solved at 3.3 Å resolution. Comparison of side-chains in a small region of the model refined on the sharpened map (green) with those of the model refined on the raw density (orange). The sharpened map density in shown in transparent grey.

We used the crystal structure of apo-GroEL (PDB: 1OEL) [59] as reference for comparing the models refined in the sharpened and raw maps. Overall, the model refined in the sharpened map is closer to the crystal structure, with Cα and all-atom RMSDs of 1.15 Å and 1.88 Å, respectively. In comparison, the raw-map based refined model has Cα and all-atom RMSDs of 1.42 Å and 2.08 Å, respectively. However, the crystal structure of the apo form does not have a perfect fit to the density throughout the chain. The apical domain (residues 190–374) of GroEL is known to be flexible and has significantly higher B-factors, compared to the equatorial domain (residues 1–135 and 410–524) [59]. The latter has the best fit in the density followed by the intermediate domain (residues 136–189 and 375–409), while the apical domain fits the worst. The model refined in the sharpened map had all-atom RMSDs of 1.49, 1.72 and 2.46 Å for the equatorial, intermediate and apical domains, respectively, from the corresponding domains in the crystal structure. On the other hand, the raw map based model had all-atom RMSDs of 1.72, 1.96 and 2.56 Å, respectively. Overall, side-chains are better refined in the sharpened map than the raw map (Fig. S5), which is expected due to the enhancement of structural features while sharpening the map. In the region involving the apical domain helices (residues 339–374), the model refined in sharpened map appears to fit better in the density, especially with respect to the backbone placement. The model refined in the sharpened map has overall better SMOC scores (calculated with the sharpened map) when compared to the model refined in the raw density. However, both models have similar SMOC profiles when scored against the raw map (data not shown).

### 3.2 Development of graphical user interface as part of CCP-EM

**Fig. 4:**
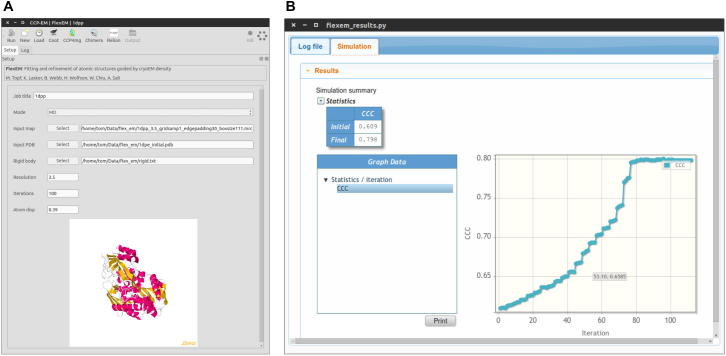
(A) Snapshot of the input parameters for Flex-EM. User can upload the electron density map, coordinates of the initial model that has to be fitted in the map and the file indicating the segments used as rigid bodies during the refinement. In order to run Flex-EM the user needs to provide the resolution of the density map and the number of iterations of Flex-EM (default is 1). (B) Snapshot of the Flex-EM results. Upon successful completion of the Flex-EM iterations, the user is provided with the plot of cross-correlation value over the different iterations, which can be used to assess the quality of fitting.

We implemented graphical user interfaces for our methods RIBFIND and Flex-EM, as part of the CCP-EM project [47]. The RIBFIND interface runs the program on an input PDB file, and the proposed RBs can be viewed interactively using a JSmol [60] widget in the results panel. The Flex-EM GUI prompts the user for an EM map, the atomic coordinates to be fitted, a file containing RB definitions from RIBFIND, and a set of useful run parameters (Fig. 4A). The job can be run locally, or submitted to a remote compute resource using the Longbow utility [61] (http://www.hecbiosim.ac.uk/wikis/index.php/Longbow). The standard output from Flex-EM and MODELLER can be viewed while the job is running. After job completion, a plot of the CCC value at the end of each iteration is displayed (Fig. 4B). In the current version, separate runs are required for the different levels of the RB hierarchy and for the different optimization methods. This allows inspection of the progress of fitting, but in the future this will be automated.

## 4 Discussion

The recent rise in the number of high resolution EM density reconstructions stirred interest in the development of methods for fitting atomic models in maps of resolution better than 5 Å. Flexible fitting approaches like MDFF, Rosetta and DireX have been applied on a few examples in this resolution range [19], [29], [62]. In this work, we demonstrated the ability of Flex-EM to refine the structure based on EM density at high resolutions. A hierarchical fitting approach was applied, where RBs reflecting protein sub-domains or SSEs are restrained to enable large body motions to fit into the density. This is followed by an all-atom refinement where the maximum atom displacement steps are reduced.

The main challenge of flexible fitting stems from the quality of the initial data. Both maps and atomic structures contain errors resulting, for example, from over-fitting of 2D images during reconstruction and from homology modelling [41] based on templates with low sequence similarity, respectively. These are difficult to quantify and can be propagated into the final models. Additionally, in sequential multi-component fitting, refinement at the interfaces can be challenging especially in cases where there is no clear segmentation between the densities of interacting partners [41]. Of course such situations can be avoided if flexible fitting of the entire complex is performed simultaneously (and in symmetric cases by applying symmetry restraints); however, this can be a challenging optimization problem, which is often computationally expensive. In the second example (Section 3.1.2) – the fitting of eIF6 into the 3.3 Å density map (EMD-3145) – we restrained the exposed loop segments at the interface in the final stages to avoid over-fitting into the density of the interacting partner.

For model building and refinement in X-ray and EM maps, density sharpening has been shown to be advantageous [58], [63], [64]. To test this with Flex-EM, we have used a new reconstruction of apo-GroEL at 3.3 Å and refined a model in both sharpened and raw maps. Indeed, comparison between the models refined in the raw map and in the sharpened map showed that the latter is closer to the crystal structure corresponding to the conformation represented by the map (PDB: 1OEL) and that the side-chains overall fit better to the density. This shows that map sharpening (e.g. using the approach from [58]) can be helpful for refinement at high resolutions. However, it may be useful to investigate different levels of sharpening on a case-by-case basis, and care should be taken to avoid over-sharpening (which can exaggerate noise and cause Fourier ripple effects [58], [64]).

Global CCC is not a useful measure to detect regions of poor fits and regions of over-fitting. In Flex-EM, the choice of RBs has a large influence on the quality of the final model. To address this problem, we used a local cross correlation score (SMOC) to assess the goodness-of-fit. SMOC profile of the starting model provides an idea on the regions that are initially fitted well and those that are poorly fitted in the density map. We previously showed that comparing local correlation profiles of structures refined by two different methods (for example Flex-EM and iMODFIT) could help to detect overfitting [41]. Here, we found that the comparison of such profiles between different stages of the refinement can highlight the improvement in local fits, which in return helps in the selection of RBs. Moreover, the SMOC profile of the refined model can also reflect segments of poor fit, when certain regions have significantly better or poorer scores than those observed on an average throughout the chain. This was achieved by calculating Z-scores based on the SMOC profile. Furthermore, comparison of this profile with the local Qmean scores corresponding to the model helped us to detect which regions could be further improved.

Finally, we showed that in some situations, like in the case of adenylate kinase (Section 3.1.1), using multiple starting conformations for local segments or loops can improve the refinement (Figs. S1 and 2(iii)) as it minimizes the requirement for a wider sampling and increases the chances of finding a local minimum in the fitting/refinement landscape [65], [66], [67]. This goes hand in hand with the idea of reducing errors through the use of multiple flexible fitting algorithms [41], [68], [69] as various algorithms suffer from different limitations in their sampling approach and scoring functions being optimized. Introducing atomic Bfactors and information on local map resolution to the process could potentially improve the results, but this is beyond the scope of this paper.

## 5 Concluding remarks

In this paper we have adjusted Flex-EM to enable the refinement of atomic models in high-resolution 3D-EM maps. Starting from various inaccurate atomic models and maps at resolutions of 2.5–4.5 Å, we demonstrated the usefulness of the protocol on a number of simulated and experimental cases, including the use of RIBFIND to identify RBs in large complexes. We are currently testing the use of both Flex-EM and RIBFIND via the CCP-EM interface that we presented here. Further, the current version of Flex-EM does not fully support nucleic acid fitting and refinement and we are testing RB definitions and optimizing the method to include these options. As discussed earlier, assessment of final models in terms of standard geometries and checks for both global and local over-fitting using approaches such as cross-validation to independently reconstructed maps and analysis of local fitting scores (as shown in this study), are crucial in density based refinement process. We will integrate some of these options for assessing the quality of the refined model, downstream to Flex-EM.

## Figures and Tables

**Table 1:** Summary of examples of Flex-EM refinement discussed in this study.

Protein	Map resolution	Initial		Final		Global CCC of X-ray or deposited model	Global CCC of refined model
Cα RMSD from X-ray or deposited model	All-atom RMSD from X-ray or deposited model	Cα RMSD from X-ray or deposited model	All-atom RMSD from X-ray or deposited model
Adenylate kinase PDB:1AKE	2.5 Å	4.53 Å	4.73 Å	0.41Å	0.96 Å	0.960	0.906
3.5 Å	0.64 Å	1.19 Å	0.985	0.907
4.5 Å	1.09 Å	1.76 Å	0.991	0.899

eIF6 EMD-3145	3.3 Å	1.90 Å	2.90 Å	1.50 Å	2.50 Å	0.720	0.741

GroEL EMD-5001	4.2 Å	6.65 Å	a	4.91 Å	a	a	0.830

a The deposited model is a Cα model.
