# Supplementary material for: Refinement of atomic models in high resolution EM reconstructions using Flex-EM and local assessment
Source: Methods. 2016 May 1;100:42–9. doi: 10.1016/j.ymeth.2016.03.007 (PMC4854230; doi:10.1016/j.ymeth.2016.03.007)
Supplement: Supplementary data 1 [file mmc1.docx]

**Protein Expression**

GroEL was kindly provided by the Horwich Lab at Yale and it’s expression and purification were done as previously described [1].

**Grid Preparation and imaging**

 3.5 μl of 4 mg/ml GroEL in 50 mM Tris-HCl (pH 7.4), 50 mM KCl, and 10 mM MgCl2 was applied to holey carbon-coated C-flat grids (r2/2, Protochips Inc., USA) and then plunge frozen. Super-resolution counting mode images were collected with EPU on an FEI Titan Krios microscope operated at 300 kV, using a slit width of 20 eV on a GIF-Quantum energy filter equipped with a K2-Summit detector. The calibrated magnification on the detector was 47,400× giving a physical pixel size of 1.055 Å (0.528 Å per super-resolution pixel). The images were recorded using a dose rate of ~8 electrons/Å^2^/s (~9 electrons/pixel/s) with 20 movie frames collected over a 4 second exposure giving a total dose of 32 electrons/Å^2^.

**Image processing**

The movies were aligned using whole-frame motion correction and then binned by 2 with an anti-aliasing filter in IMOD [2]. The Contrast transfer function for each aligned movie was determined using CTFFIND3 and all subsequent processing steps were performed in RELION-1.3 [3, 4]. References for template-based particle picking were generated from 2D class averages that were calculated from a manually picked subset of the micrographs. The 34,500 Selected particles were then classified in 2D with only the best 17,400 used for 3D classification and refinement. a 60Å low-pass filtered reconstruction of GroEL was used as a reference for 3D classification, with a 20Å low-pass filtered reconstruction from 3D classification used as a reference for 3D refinement. The resolutions was calculated to be 3.26 Å using the gold-standard refinement procedures and the corresponding FSC=0.143 criterion [5]. The density map was then sharpened by applying a negative B-factor that was estimated using automated procedures [6].

**Acknowledgements**

We would like to thank Diamond for access to the cryo-EM facilities at the UK national electron bio-imaging centre and S. Welsch and A. Seibert for help with data collection.

[1] H.S. Rye, et al., Nature, 388 (1997) 792-798.

[2] J.R. Kremer, D.N. Mastronarde, J.R. McIntosh, Journal of structural biology, 116 (1996) 71-76.

[3] J.A. Mindell, N. Grigorieff, Journal of structural biology, 142 (2003) 334-347.

[4] S.H. Scheres, Journal of structural biology, 180 (2012) 519-530.

[5] S.H. Scheres, S. Chen, Nature methods, 9 (2012) 853-854.

[6] P.B. Rosenthal, R. Henderson, Journal of molecular biology, 333 (2003) 721-745.
